# Global Civil Unrest: Contagion, Self-Organization, and Prediction

**DOI:** 10.1371/journal.pone.0048596

**Published:** 2012-10-31

**Authors:** Dan Braha

**Affiliations:** 1 New England Complex Systems Institute, Cambridge, Massachusetts, United States of America; 2 University of Massachusetts, Dartmouth, Massachusetts, United States of America; 3 Massachusetts Institute of Technology, Cambridge, Massachusetts, United States of America; University of Zaragoza, Spain

## Abstract

Civil unrest is a powerful form of collective human dynamics, which has led to major transitions of societies in modern history. The study of collective human dynamics, including collective aggression, has been the focus of much discussion in the context of modeling and identification of universal patterns of behavior. In contrast, the possibility that civil unrest activities, across countries and over long time periods, are governed by universal mechanisms has not been explored. Here, records of civil unrest of 170 countries during the period 1919–2008 are analyzed. It is demonstrated that the distributions of the number of unrest events per year are robustly reproduced by a nonlinear, spatially extended dynamical model, which reflects the spread of civil disorder between geographic regions connected through social and communication networks. The results also expose the similarity between global social instability and the dynamics of natural hazards and epidemics.

## Introduction

Civil unrest contagion occurs when social, economic, and political stress accumulate slowly, and is released spontaneously in the form of social unrest on short time scales to nearest and long-range neighboring regions that are susceptible to social, economic, and political stress [1]–[5]. Unrest events have led to significant societal and cultural changes throughout history. Examples include the spread of discontent in France in 1848 that proliferated to most of Europe and parts of Latin America; the wave of urban racial riots in the United States in the 1960s; and the 1989 uprisings against communism in various central and eastern European countries, symbolized by the fall of the Berlin Wall. More recently, social instability has spread rapidly in the Arab world – from nonviolent protest movements in Tunisia and Egypt that toppled long-established authoritarian regimes, to a protest movement that evolved to a full-blown civil war in Libya. These social unrest events span the full spectrum from civil wars, revolutions, and coups d’état that have killed millions of people to relatively peaceful forms of intra-state conflicts, such as anti-government demonstrations, riots, and general strikes [5]–[10].

**Figure 1:**
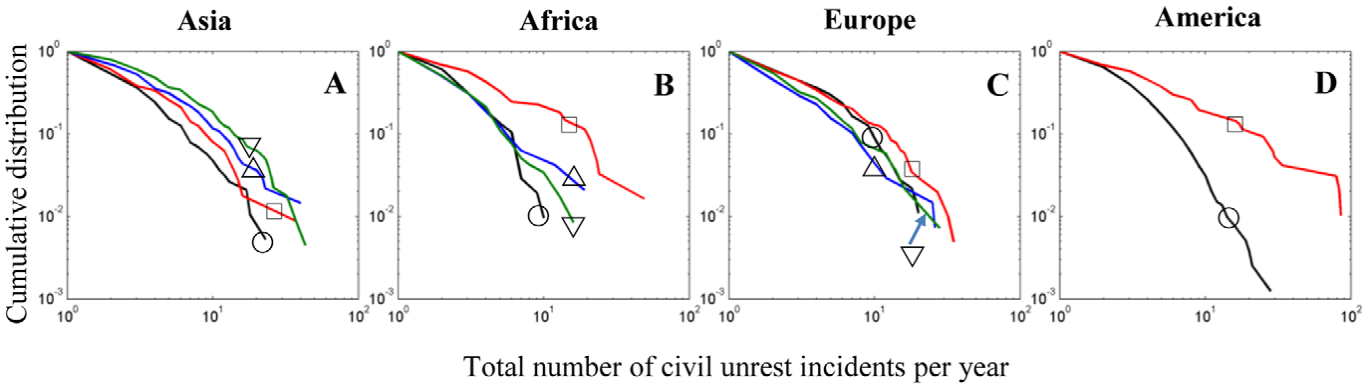
Observed civil unrest event count distributions. The incidence of civil unrest events per year is measured by summing over the reported country-level number of anti-government demonstrations, riots, and general strikes (see the section Domestic Conflict Data), for all countries within a particular subregion of the world (see the section Countries and Geographical Regions Included in the Study). The figure shows the log-log plot of the complementary cumulative distribution of civil unrest event count, 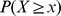.(**A**) Unrest event count distributions for geographical subregions of Asia: Western Asia (○); South-Eastern Asia (□); Eastern Asia (▵); Southern Central Asia (▿). (**B**) Unrest event count distributions for geographical subregions of Africa: Western Africa (○); Southern Africa (□); Middle Africa (▵); Eastern Africa (▿). (**C**) Unrest event count distributions for geographical subregions of Europe: Western Europe (○); Southern Europe (□); Northern Europe (▵); Eastern Europe (▿). (**D**) Unrest event count distributions for geographical subregions of America: Caribbean, Central, and South America (○); North America (□).

A pertinent question from large-scale social dynamics and policymaking standpoints is what causes the extent and outbreaks of civil unrest spreading. Social unrest has been attributed to a variety of social, political, economic, and environmental causes including racial and ethnic tensions [11], food scarcity and food price increases [6]–[10], variations in international commodity prices [12], [13], economic shocks [14], climate change and rainfall shocks [15], [16] and demographic changes [17]. Despite these conditions, it is shown that external causes are not necessary to explain the observed magnitude of almost a century of riots and collective protests across the world. Instead, a parsimonious explanation of social unrest dynamics is provided based on a hypothesis that widespread unrest arises from internal processes of positive feedback and cascading effects in the form of contagion and social diffusion over spatially interdependent regions connected through social and mass communication networks. Here, records of civil unrest events are analyzed, compiled from newspaper reports, of 170 countries covering the period of 1919 through 2008 (see the section Domestic Conflict Data for details). The long-term event dataset analyzed here includes the number of incidents of three main indicators: anti-government demonstrations, riots, and general strikes. Countries are grouped by their geographical region (see the section Countries and Geographical Regions Included in the Study), and the corresponding distributions of the number of civil unrest events per year are studied. The results reported here (described later in the section Results and Discussion) also apply at the level of individual countries. Fig. 1 shows the size distributions of the number of unrest incidents (the sum over the three event indicators) for various geographical regions of the world. The data indicates that there is a wide variation in the characteristics of civil unrest with no apparent pattern of unrest dynamics in time or geographical space. Although the study of collective human dynamics, including collective aggression, has been the focus of much discussion in the context of modeling and identification of universal patterns of behavior [18]–[25], the mechanisms leading to this diverse behavior of social unrest are unclear, and have never been attempted before. Here, social instability is considered as a generalized spatial epidemics phenomenon, similar to other spatially extended dynamical systems in the physical and biological sciences, such as earthquakes, forest fires, and epidemics [26]–[28]. The model presented below provides a parsimonious quantitative framework that is able to explain and reproduce the full range of empirical civil unrest event count distributions for all regions as shown in Fig. 1.

## Model

**Figure 2:**
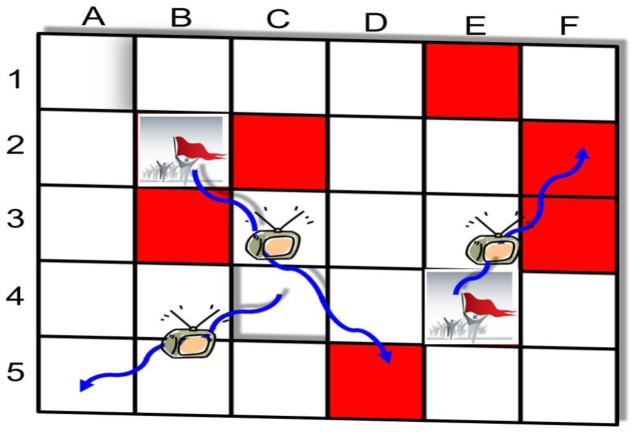
Social unrest spatial contagion model. The model is defined on a square grid of 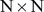 sites, which represents the division of a country into urban clusters (see the section Parameter Estimation for details). Each site of the grid can be in one of three different states: empty (“white”), susceptible to social unrest (“red”), and involved in social unrest (“crowd”). Sites are one grid step apart if they are directly adjacent to each other in either the horizontal or vertical direction, or are connected through weak links (e.g., site E4 is connected to site F2). The weak links are formed by associating with each site, with probability 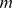, a single link to a site selected uniformly at random from the grid. The grid is updated synchronously according to the following rules: at each time step, empty sites become susceptible with probability 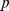, and susceptible sites become involved in social unrest with probability 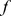. Unrest contagion occurs on a short time scale as follows. If a site is involved in social unrest (e.g., sites B2 and E4), the unrest activity spreads with probability 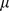 to susceptible sites that are one grid step apart (e.g., sites C2, B3, or D5), which in turn can lead (with probability 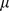) to further instabilities of susceptible sites that are two grid steps apart, three grid steps apart, and so on. Each of the sites involved in social unrest during a time step contributes to the size of the unrest contagion.

The model (shown schematically in Fig. 2) divides the country into sites (“urban clusters”) placed on a two-dimensional grid. It is assumed that unrest activity is transmitted, with infectiousness probability 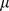, along two kinds of links: short-range links between sites that are directly adjacent to each other in either the horizontal and vertical directions; and long-range links created with probability 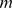 between each site and another site selected uniformly at random from the grid. The connectedness between sites on the grid reflects geographic proximity, social proximity, and proximity within the mass communication networks along which social instability is transmitted (the effect of mass media distribution networks on the pattern of unrest activity is examined in the section Telecommunication Technologies and Social Unrest). The overlay network construction is similar to the small-world model [29], although the values of the fitted parameter 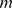 will be found to deviate from the small world regime (see the section Results and Discussion). An alternative way for modeling the distribution of long-range links is explored in the section The Effect of Network Structure on Social Unrest. Social, economic, and political stress accumulates slowly on the grid with probability 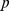 per site (‘unrest susceptibility’ rate), which then become susceptible to unrest activity. Social unrest is released spontaneously with probability 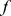 per susceptible site (‘spontaneous outburst’ rate), and is diffused on short time scales to nearest and distant susceptible sites. This activity can lead to further instabilities and avalanches of unrest events throughout the grid. The simulated incidence of civil unrest is measured by the number of sites that are involved in the spread of unrest activity.

**Figure 3:**
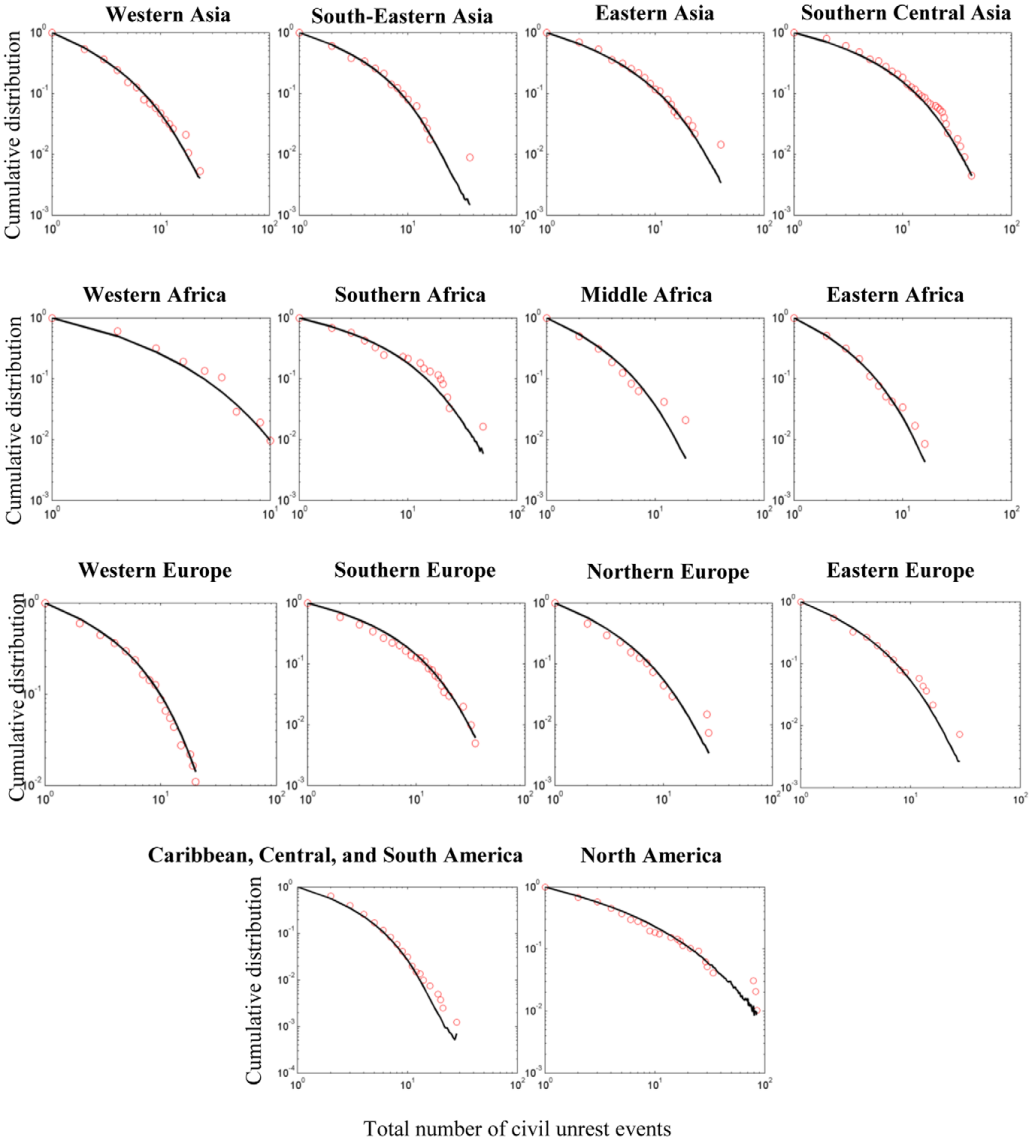
Observed data and best-fit curves for civil unrest event count distributions. Observed values are denoted by circles. Solid best-fit lines denote average distributions calculated from 500 realizations of the social unrest contagion model. The goodness of fit of the model relative to the empirically observed unrest event count distributions was determined by measuring the distance between the observed and simulated distributions. Here, the tail-weighted Kolmogorov–Smirnov (wKS) statistic is used (see the section Assessing the Goodness of Fit of the Model). The fit of the model is very good for all regions. Goodness-of-fit (wKS) and fitted parameters 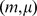 for all regions: Western Asia = 0.1269 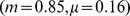, South-Eastern Asia = 0.1581 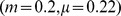, Eastern Asia = 0.1492 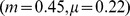, Southern Central Asia = 0.1078 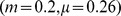, Western Africa = 0.1749 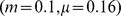, Southern Africa = 0.2513 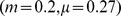, Middle Africa = 0.1856 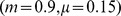, Eastern Africa = 0.1191 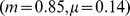, Western Europe = 0.1436 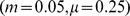, Southern Europe = 0.11220 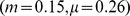, Northern Europe = 0.16 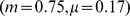, Eastern Europe = 0.1634 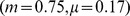, Caribbean, Central, and South America = 0.0707 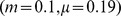, North America = 0.2349 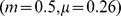. Values of wKS that are less than 0.3 represent good fits (see the section Assessing the Goodness of Fit of the Model).

## Results and Discussion

A plausible size for the grid is specified based on changes in the average population of a country over the period analyzed (1919 to 2008), and a characteristic urban cluster as defined by the U.S. Census Bureau (see the section Parameter Estimation for details). The outburst 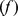 and susceptibility 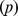 rates have been set such that 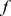
≪
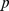
≪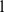, which reflects the time scale separation that often underlies riots, unrest and revolutions [5]–[10]. This leaves only two free parameters: the probability 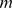 of establishing long-range links between sites, and the infectiousness rate 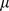 of transmitting social instability on short time scales to nearest and distant susceptible sites. Given a set of parameters, a computer simulation was run for a number of time steps (see the section Parameter Estimation), and the simulated distribution of the total unrest event count was determined. The free parameters associated with specific geographical regions of the world were chosen by minimizing the statistical distance between the simulated and empirical distributions (see the section Assessing the Goodness of Fit of the Model). Using these parameters in the computer simulation model, it is found that the model is able to reproduce the observed distributions remarkably well over the full range of the world’s geographic regions (see Fig. 3).

**Figure 4:**
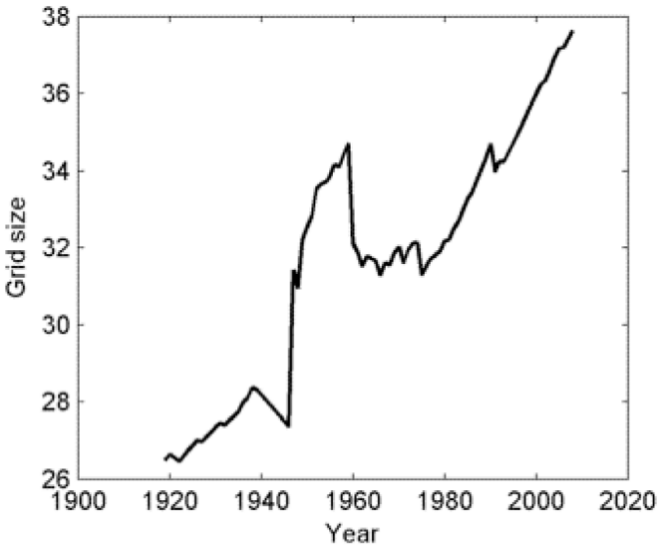
Calculated size of the grid over time based on an average urban cluster of 26250 people.

**Figure 5:**
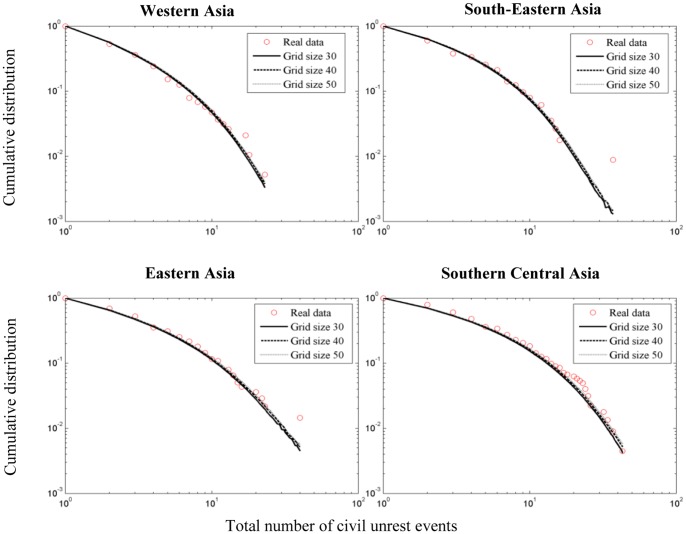
Goodness-of-fit (average wKS statistics) and simulated event count distributions for various grid sizes. Observed values for each sub-region of Asia are denoted by circles. Solid best-fit lines denote average distributions calculated from 500 realizations of the social unrest contagion model by using a grid size 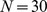. Simulated count distributions for grid sizes 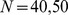 use the fitted parameters obtained for 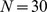 (see Fig. 3). Goodness-of-fit (wKS) for all regions and grid sizes: Western Asia: 0.1269 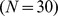, 0.1254 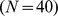, 0.1265 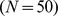; South-Eastern Asia: 0.1581 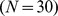, 0.1682 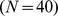, 0.1692 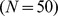; Eastern Asia: 0.1492 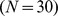, 0.1521 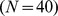, 0.1598 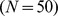; Southern Central Asia: 0.1078 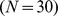, 0.1161 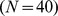, 0.1199 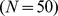. The differences between the curves are too small to be visually noticeable. Running time on an Intel i7 Core processor was approximately 6.6 hours 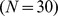, 21 hours 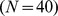, 50.56 hours 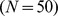.

The unrest contagion model is sufficiently flexible to accommodate a wide range of possible unrest event count distributions. “Broad-scale” distributions that show a power–law regime with a sharp cutoff in the tail are obtained when the infectiousness rate 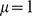, and when the outburst rate 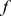 is very small relative to the susceptibility rate 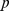 (i.e., 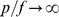), even in the absence of long-range connections 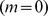. “Single-scale” distributions with fast-decaying tails arise when the infectiousness rate 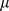
≪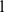, in which case augmenting the grid with a network of long-range connections 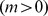 could lead to broad-scale distributions. In general, different forms of social or communication networks that connect the regions of the country will generate different civil unrest event count distributions (see the section The Effect of Network Structure on Social Unrest where the effect of heterogeneous network structures on civil unrest spreading is analyzed).

**Figure 6:**
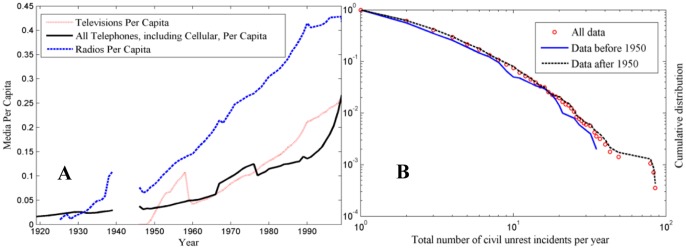
Effect of mass media on civil unrest activity. (**A**) Change in the total number of radios, television receivers, and phones, owned or operated per capita: 1919 to 2008. Widespread use of telecommunication technologies started around 1950. (**B**) The empirically observed distributions of the number of unrest incidents in the world: for the entire period of 1919–2008, and for pre- and post-1950.

The unrest contagion patterns of each of the world’s geographic regions (Fig. 1) are uniquely characterized by the parameters of the model (Fig. 3). Unlike critical phenomena where universality arises from the irrelevance of particular details of the system [30], here universality arises from the fact that social unrest contagion is governed by the same mechanisms despite idiosyncrasies of individual countries and geographic regions. The mechanisms uncovered separate the phenomenon of rioting and social instability into three time scales: the unrest infectiousness rate from disrupted regions to neighboring regions that are susceptible to social unrest, the rate by which regions become susceptible to unrest activity due to social, economic, and political stress, and the rate by which social unrest is released spontaneously in susceptible regions (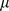
≫
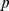
≫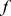). The spatial contagion mechanism here arises from interdependence of closely related regions; people participate in collective protest because of long-standing social, economic, and political stress, and because others have recently done so. If rioters see others they might respond similarly even if their external conditions have not changed, and protests spreads across social networks and from place to place. While the presented parsimonious model does not prove that exogenous causes play no role in determining the intensity of civil unrest, it does say that exogenous causes are not necessary to explain the observed data, and that the pursuit of independent variables that predict the occurrence of civil unrest events in space and time may be illusory.

**Figure 7:**
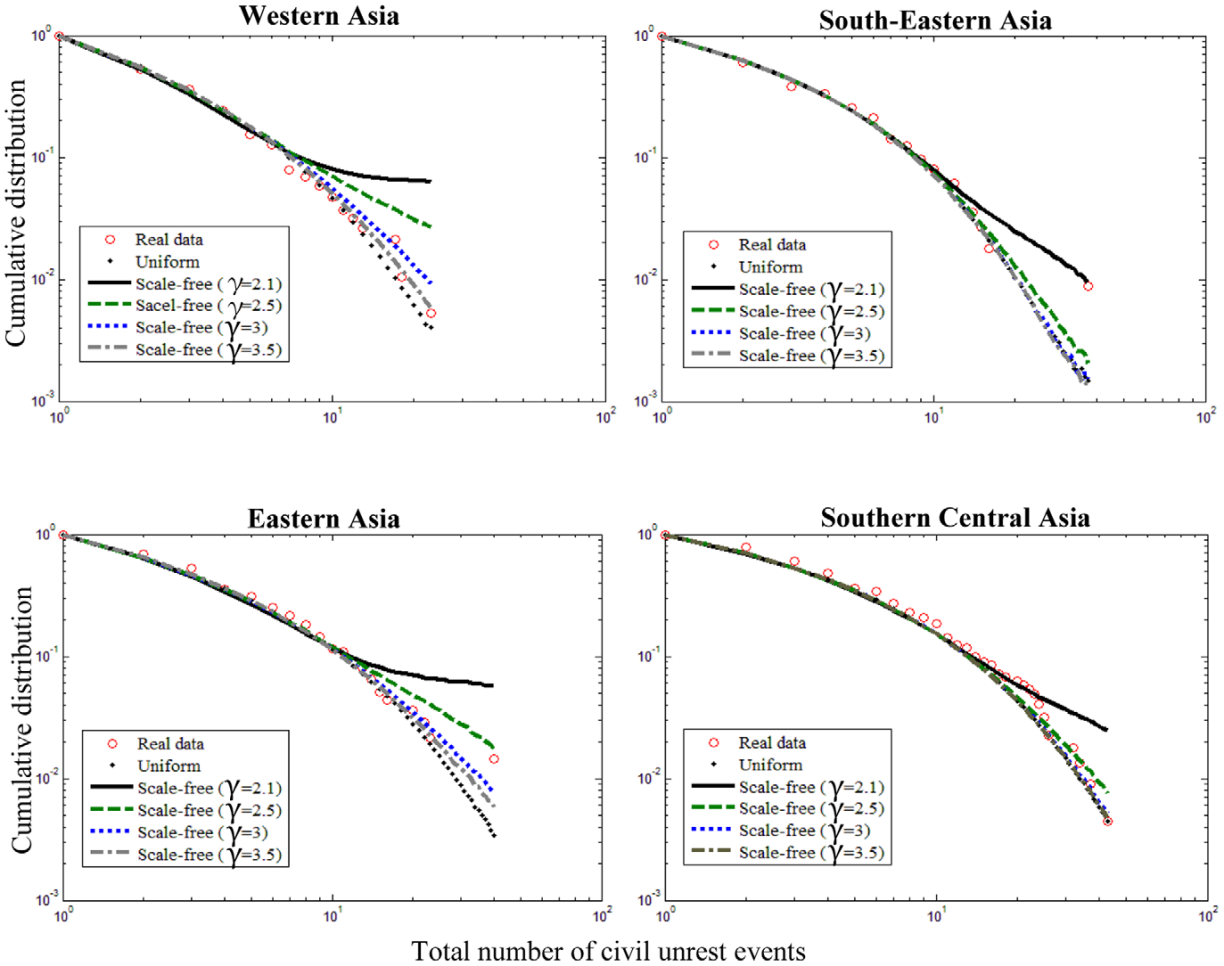
Effect of network structure on civil unrest activity. Observed values for each sub-region of Asia are denoted by red circles. Black points denote best-fit average distributions calculated from 500 realizations of the social unrest contagion model by using a grid size 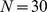 and a uniform distribution of long-range links (fitted parameters 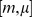 for all regions are given in Figure 3). In all cases, a scale-free overlay network was generated with roughly the same number of directed links, 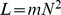, as in the corresponding uniform overlay network. Here, 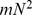 is the expected number of long-range links on a square grid of 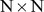 sites when the uniform distribution is used.

The reported results have several practical implications. First, the parameters of the model can be estimated from unrest data that includes small and medium sized events, and then be used to quantify the risk of large-sized events. Second, monitoring the parameters of the model and trends in their values over time through comprehensive ongoing unrest data, may serve as an early warning signal for increased vulnerability to social instability.

## Materials and Methods

### Domestic Conflict Data

This research utilizes a long-term dataset, which traces out several indicators of domestic conflict in 170 countries and covers the years from 1919 to 2008. These data are part of the comprehensive Cross National Time Series Dataset [31]. The main source of data on unrest incidents are reports of the New York Times. The three main indicators of domestic conflict included in the analysis are general strikes, riots, and anti-government demonstrations, which are defined as follows [31], [32]: General Strikes – any strike of 1,000 or more industrial or service workers that involves more than one employer and that is aimed at national government policies or authority; Riots – any violent demonstration or clash of more than 100 citizens involving the use of physical force; Anti-government Demonstrations – any peaceful public gathering of at least 100 people for the primary purpose of displaying or voicing their opposition to government policies or authority, excluding demonstrations of a distinctly anti-foreign nature. It is expected that the likelihood of a social unrest event being reported in the media will increase with the number of people involved in the social unrest activity. Thus, focusing on large-magnitude social unrest events reduces the effect of various reporting biases. The database includes 1046 (Africa), 2912 (Asia), 2380 (Europe), and 3117 (America) unique social unrest events.

### Countries and Geographical Regions Included in the Study

The countries and geographical regions included in this study are based on the United Nations geographical region classification [33]. Several dependent territories or geographical regions, for which data was not available or sufficiently detailed to allow a reliable comparison with model predictions, were excluded from the study.

#### Eastern Africa

Burundi, Comoros, Djibouti, Eritrea, Ethiopia, Kenya, Madagascar, Malawi, Mauritius.

Mozambique, Rwanda, Seychelles, Somalia, Uganda, United Republic of Tanzania, Zambia, Zimbabwe.

Middle Africa: Angola, Cameroon, Central African Republic, Chad, Congo, Democratic Republic of the Congo, Equatorial Guinea, Gabon, Sao Tome and Principe.

#### Southern Africa

Botswana, Lesotho, Namibia, South Africa, Swaziland.

#### Western Africa

Benin, Burkina Faso, Cape Verde, Cote d’Ivoire, Gambia, Ghana, Guinea, Guinea-Bissau, Liberia, Mali, Mauritania, Niger, Nigeria, Senegal, Sierra Leone, Togo.

#### Southern Central Asia

Kazakhstan, Kyrgyzstan, Tajikistan, Turkmenistan, Uzbekistan, Afghanistan, Bangladesh, Bhutan, India, Iran (Islamic Republic of), Maldives, Nepal, Pakistan, Sri Lanka.

#### Eastern Asia

China, Democratic People’s Republic of Korea, Japan, Mongolia, Republic of Korea.

#### South-Eastern Asia

Brunei Darussalam, Cambodia, Indonesia, Lao People’s Democratic Republic, Malaysia, Myanmar, Philippines, Singapore, Thailand, Timor-Leste, Viet Nam.

#### Western Asia

Armenia, Azerbaijan, Bahrain, Cyprus, Georgia, Iraq, Israel, Jordan, Kuwait, Lebanon, Oman, Qatar, Saudi Arabia, Syrian Arab Republic, Turkey, United Arab Emirates, Yemen.

#### Eastern Europe

Belarus, Bulgaria, Czech Republic, Hungary, Poland, Republic of Moldova, Romania, Russian Federation, Slovakia, Ukraine.

#### Northern Europe

Denmark, Estonia, Finland, Iceland, Ireland, Latvia, Lithuania, Norway, Sweden, United Kingdom of Great Britain and Northern Ireland.

#### Southern Europe

Albania, Andorra, Bosnia and Herzegovina, Croatia, Greece, Italy, Malta, Montenegro, Portugal, San Marino, Serbia, Slovenia, Spain, The former Yugoslav Republic of Macedonia.

#### Western Europe

Austria, Belgium, France, Germany, Liechtenstein, Luxembourg, Monaco, Netherlands, Switzerland.

#### Caribbean

Antigua and Barbuda, Aruba, Bahamas, Barbados, Cuba, Dominica, Dominican Republic.

Grenada, Haiti, Jamaica, Saint Kitts and Nevis, Saint Lucia, Saint Vincent and the Grenadines, Trinidad and Tobago.

#### Central America

Belize, Costa Rica, El Salvador, Guatemala, Honduras, Mexico, Nicaragua, Panama.

#### South America

Argentina, Bolivia (Plurinational State of), Brazil, Chile, Colombia, Ecuador, Guyana Paraguay, Peru, Suriname, Uruguay, Venezuela (Bolivarian Republic of).

#### Northern America

Canada, United States of America.

### Assessing the Goodness of Fit of the Model

The output of the social unrest contagion simulation model is a set of unrest event counts. The goodness of fit of this set relative to the empirically observed unrest count distribution was determined by measuring the distance between the observed and simulated distributions. Here, the tail-weighted Kolmogorov–Smirnov (wKS) statistic is used [34]. The wKS statistic is a variant of the more commonly used Kolmogorov–Smirnov (KS) goodness-of-fit statistic [35], which is defined as the maximum distance between the cumulative distribution functions of the observed data and the fitted simulation model. It is well known that the Kolmogorov-Smirnov (KS) statistic exhibits poor sensitivity to deviations from the hypothesized distribution, which occur in the tails [36]. The modified wKS statistic gives equal weight to all parts of the distribution and particularly to the tails than does the KS statistic. More specifically, given the set of observed unrest size data, a cumulative distribution function 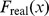 is computed. The cumulative distribution function 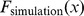 is also computed, given the set of unrest size data taken from the simulation results of the social unrest contagion model. The tail-weighted Kolmogorov–Smirnov (wKS) statistic is then defined as
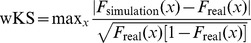
(1)Values that are very small 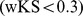 indicate a strong similarity between the observed and simulated distributions [34].

### Parameter Estimation

In this section the methods used to calibrate the social unrest contagion model shown in Fig. 2 are described.

#### Size of the grid

The social unrest contagion model is defined on a square grid of 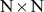 sites, which represents the division of a country into urban clusters. Urban areas often tend to pose serious problems with respect to ecosystem services, poverty, and human well-being [37], which are inextricably linked with social unrest [6]–[10], [37]. Administrative and population-based criteria of urban areas are different in different countries and across time [37]. Here, the definition of urban areas used by the U.S. Census Bureau is utilized. It has two distinct categories: “urbanized areas” have populations of greater than 50,000, while “urban clusters” have populations of at least 2,500 and less than 50,000 people [38]. Long-term population data of all countries analyzed, covering the years from 1919 to 2008, are obtained [31]. Let 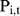 denote the population size of country i at year t, and let 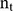 be the total number of countries at time t. The average size of the grid at year t is then defined as
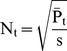
(2)where 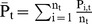 is the average country population at year t, and s is a characteristic size of an urban cluster. Assuming an average urban cluster of 26250 people, Figure 4 shows the average size of the grid versus time. It is seen that the calculated size of the grid over time varies between 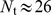 and 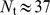. In the simulations, a value of 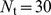 is considered as a plausible scenario. Indeed, it is observed that the goodness-of-fit procedure (described in the section Assessing the Goodness of Fit of the Model) is not overly sensitive to the choice of larger 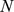. To illustrate, average wKS statistics (average distance between real and simulated data, see ‘Simulations and data fitting’ below for details) and event count distributions for grid sizes 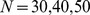 are compared. Figure 5 shows the results for the sub-regions of Asia. It is noted that while the computational time increases significantly with the size of the grid, the average wKS values (see ‘Simulations and data fitting’ below for details) and event count distributions are not appreciably altered by the size of the grid.

#### Spontaneous outburst and susceptibility rates

The spontaneous outburst 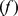 and susceptibility 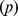 rates have been set such that 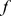
≪
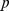
≪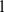, which reflects the time scale separation that often underlies riots, unrest and revolutions. In other words, it is assumed that social, economic, and political tensions accumulate slowly throughout the country before they lead to sudden outbursts of unrest and ensuing contagious turmoil events [6]–[10]. Given the annual frequency of reported social unrest data, it is expected that ∼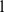 sudden unrest events to occur each year. The slow accumulation of social, economic, and political tensions is operationalized by letting ∼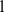 new susceptible regions to appear each day. In the simulations, I set 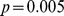 and 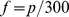, which approximately satisfy these assumptions.

#### Simulations and data fitting

The model was fitted to civil unrest data for each individual geographical region of the world using the tail-weighted Kolmogorov–Smirnov (wKS) statistic as described in the section Assessing the Goodness of Fit of the Model. The model has two free parameters: the probability 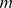 of establishing long-range links between sites, and the infectiousness rate 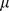 of transmitting social instability on short time scales to nearest and distant susceptible sites. Since it is computationally impractical to search for the optimal parameters, the approach used here was to lay a 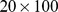 grid over the entire parameter space, which gives an accuracy of 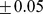 and 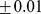 for the optimal parameters 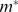 and 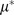, respectively. Finer-scale steps for the parameter 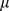 are used since the wKS statistic is more sensitive to changes in 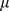 than changes in 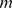. For each pair of parameters 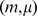, the average distance (wKS statistic) between the observed and simulated event count distributions is calculated from 500 repetitions of the simulation (taking about 6.6 hours of Intel i7 Core processor time to complete), which gives a relative error of a few percent with a probability of 0.95. Each repetition of the simulation generated 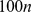 events, where 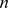 is the number of countries within the geographical region. Finally, the optimal pair of parameters that minimizes the average wKS statistic over the entire parameter space was selected. The best-fit curves for the various geographical regions of the world are shown in Fig. 3. In all of the cases considered here, the best-fit curves are in remarkable agreement with observed unrest count distributions 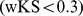.

### Telecommunication Technologies and Social Unrest

Social unrest diffusion is often transmitted by some sort of a communication network [39]–[41]. Along with printed newspapers, the invention of the telegraph has immediately become an important tool for the transmission of news around the world circa 1848. Similarly, the use of radio and television receivers has made the unrest influence among cities not only by the geographic location of cities, but also by proximity within the mass media distribution networks. For example, the mass media have played a crucial role in the spread of the 1960s riots in the United States. More recently, social networking websites such as Facebook, YouTube and Twitter have helped spread civil unrest news events and social influence quickly around the globe. For example, González-Bailón et al [41] have analyzed Twitter activity of recruitment around the protests that took place in Spain in May 2011, and have reported evidence of social influence and complex contagion.

In this section, the effect of mass media distribution networks on the patterns of unrest activity is examined. Long-term population data, number of radio receivers, television receivers, and all telephones (including cellular) of all countries analyzed, covering the years from 1919 to 2008, are obtained [31]. For each media type, the per capita rate for each year was then calculated by summing the total number of units within a year across all countries, and dividing the sum by the total population of all countries. Figure 6A shows the rapid increase in the total number of radios, television receivers, and phones, owned or operated per capita since circa 1950. The interplay between the rapid increase in telecommunication technologies over the past century and global civil unrest is illustrated in Figure 6B, which shows the size distributions of the number of unrest incidents in the world before and after the widespread use of radio, television receivers, and phones. We notice that the unrest event count distributions (before and after 1950) are very similar in the low unrest region up to a certain value, where they start to deviate. The results could be explained in terms of the unrest contagion model presented in the section Model: the left side of the distribution, corresponding to small and medium number of unrest incidents, is mainly associated with unrest activity that is transmitted between local, geographically close regions; while the distribution becomes skewed to the right mostly due to unrest activity that is spread through the mass media to regions that are not necessarily contiguous. In this context, Figure 6B shows that, despite the rapid increase of telecommunication technologies over time, the size distribution of the number of unrest incidents for the entire period of 1919–2008 captures the essence of the patterns shown for pre- and post-1950, thus confirming the robustness of the unrest contagion mechanism and results presented in the section Results and Discussion.

### The Effect of Network Structure on Social Unrest

As discussed in the section Telecommunication Technologies and Social Unrest, civil unrest may spread through media networks or through social networks where protesters contact their recruits in other cities, encouraging them to join a protest. In the section Model, this unrest spreading process was modeled by overlaying a small-world network on top of a two-dimensional grid. In this model, two kinds of links are considered: short-range links between sites that are directly adjacent to each other; and long-range links selected uniformly among all sites. While the short-range links capture the geographical nature of civil unrest spreading, a plausible alternative to the uniform distribution of long-range links would be to consider a more heterogeneous distribution. Here we are motivated by major advances that have been made in understanding the structure and dynamics of real-world social, biological, and technological complex networks [42]–[44]. Of particular interest are scale-free networks where the degree (i.e., the number of nodes adjacent to a node) is distributed according to a power law or a long right tail distribution, implying the existence of highly connected nodes called hubs [45]. The long-tail characterization of complex networks has helped in understanding and predicting the behavior of social, biological, and technological networked systems, including their robustness against failures [46], [47], vulnerability to deliberate attacks [46], [48], and more relevant to our case here, epidemic spreading and diffusion properties [49], [50].

Here, the role of fat-tailed distributions of long-range links in civil unrest spreading is analyzed. To this end, the small-world construction of the section Model is modified by replacing the uniform distribution of long-range links with a procedure in which the grid is augmented by a set of links randomly chosen from a power-law distribution. In particular, directed scale-free networks with different exponents were generated using the static model presented in [51]. For a square grid of 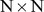 sites with 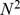 sites (‘vertices’), the sites are indexed by an integer 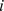 (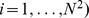, and a weight 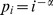 to each site is assigned, where 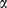 is a tunable parameter in 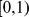. Next, two different sites 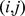 are selected with probabilities 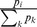 and 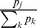, respectively. A directed link 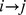 is then added between them if none already exists. This process is repeated until 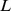 directed links are added to the grid. This procedure robustly generates [51] a directed scale-free network in which both the in-degree and out-degree distributions follow the power law 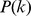
∼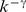, where 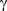 is given by 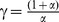. By varying the parameter 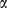, one can obtain scale-free networks with different exponents 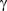 in the range 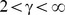. The above procedure can be easily modified (with two control parameters) to account for the case where the in-degree and out-degree follow power laws with different exponents [51], but the above procedure will do for our purpose here.

We have tested the effect of various network topologies on civil unrest spreading. The computer simulations include: two-dimensional grids with a uniform distribution of long-range links as described in the section Model, and two-dimensional grids with a power law distribution of long-range links. For comparison, the scale-free networks were generated with roughly the same number of directed links as the uniform overlay networks (see Figure 7 for details). Figure 7 shows that while the uniform and scale-free overlay networks generate unrest event count distributions that are very similar (almost coincide) in the low unrest region up to a certain value, the distributions start to deviate from each other and from the observed unrest data for large event sizes. With respect to the power-law exponent 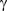, the smaller the exponent, the more high-degree sites are present in the grid, which increases the effect of large unrest event sizes. A possible direction of future work is to compare the performance of various overlay network topologies (using the methods presented in the sections Assessing the Goodness of Fit of the Model and Parameter Estimation), including the scale-free and uniform structures discussed above.
